# Identification of novel SARS-CoV-2 3CLpro inhibitors by molecular docking, *in vitro* assays, molecular dynamics simulations and DFT analyses

**DOI:** 10.3389/fphar.2024.1494953

**Published:** 2024-10-30

**Authors:** Keli Zong, Chaochun Wei, Wei Li, Jiajun Ruan, Susu Zhang, Jingjing Li, Xiaojing Liu, Xu Zhao, Ruiyuan Cao, Hong Yan, Xingzhou Li

**Affiliations:** ^1^ College of Chemistry and Life Science, Beijing University of Technology, Beijing, China; ^2^ Beijing Institute of Pharmacology and Toxicology, Beijing, China; ^3^ Department of Hepatology, Fifth Medical Center of Chinese PLA General Hospital, Beijing, China

**Keywords:** 3CLpro, molecular docking, enzymatic assay, molecular dynamics, DFT

## Abstract

**Introduction:**

SARS-CoV-2 pandemic has presented a significant threat to global health and the economy, necessitating urgent efforts to develop effective antiviral drugs. The main protease (3CLpro) of SARS-CoV-2 is a critical target for antiviral therapy due to its essential role in viral replication.

**Methods:**

In order to find new structural types of 3CLpro inhibitors to facilitate the solution to the problem of new virus resistance. Six potential pharmacologically bioactive compounds were identified by utilizing structure-based virtual screening and *in vitro* assays from the Topscience database containing 10 million compounds.

**Results and Discussion:**

Among these, compounds 34 and 36 exhibited potent inhibitory activity with IC_50_ values of 6.12 ± 0.42 μM and 4.47 ± 0.39 μM, respectively. To elucidate their binding mechanisms with 3CLpro, all-atom molecular dynamics (MD) simulations were conducted. Principal component analysis (PCA), free energy landscapes (FEL) and dynamic cross-correlation maps (DCCM) revealed that the binding of compounds 34 and 36 to 3CLpro significantly enhanced the structural stability of 3CLpro, reducing conformational flexibility and internal motions. The results of protein-ligand interaction showed that compounds 34 and 36 formed strong and stable interactions to key residues at active site of 3CLpro with different binding modes from S-217622. And HOMO-LUMO gap and molecular electrostatic potential distribution revealed the quantum chemical properties of compounds 34 and 36. These findings suggested that compounds 34 and 36 can be as novel SARS-CoV-2 3CLpro inhibitors and promising lead-like drug candidates for developing COVID-19 treatments.

## 1 Introduction

The global COVID-19 pandemic caused by the new severe acute respiratory syndrome coronavirus 2 (SARS-CoV-2) has resulted in more than 500 million confirmed illnesses and nearly 6.9 million deaths worldwide (Mozaffari et al., 2022; Pillai et al., 2024). Following its emergence in late 2019, SARS-CoV-2 continuously evolved into several new variants, including Alpha (B.1.1.7), Beta (B.1.351), Gamma (P.1), Delta (B.1.617.2), and Omicron (B.1.1.529). These variants were designated as variants of concern because of their heightened transmission efficiency. The latest variant JN.1 (BA.2.86.1.1) (Kaku et al., 2024; Young et al., 2021) has been classified as a variant of interest. Although the globe has resumed its regular state after the pandemic, the current measures to prevent and treat the virus were insufficient in effectively dealing with the difficulties it presents. Severe cases and deaths still occurred from time to time (Cortegiani et al., 2021). Due to viral mutations and the inherent limitations of current vaccines, the existing vaccines have been unable to fully prevent the spread of SARS-CoV-2 (Dutta and Iype, 2021). Antiviral drugs had the potential to prevent SARS-CoV-2 from advancing to severe illness and facilitated faster viral clearance in patients, thereby lowering transmission rates, which positioned them as a vital tool in combating SARS-CoV-2.

**FIGURE 1:**
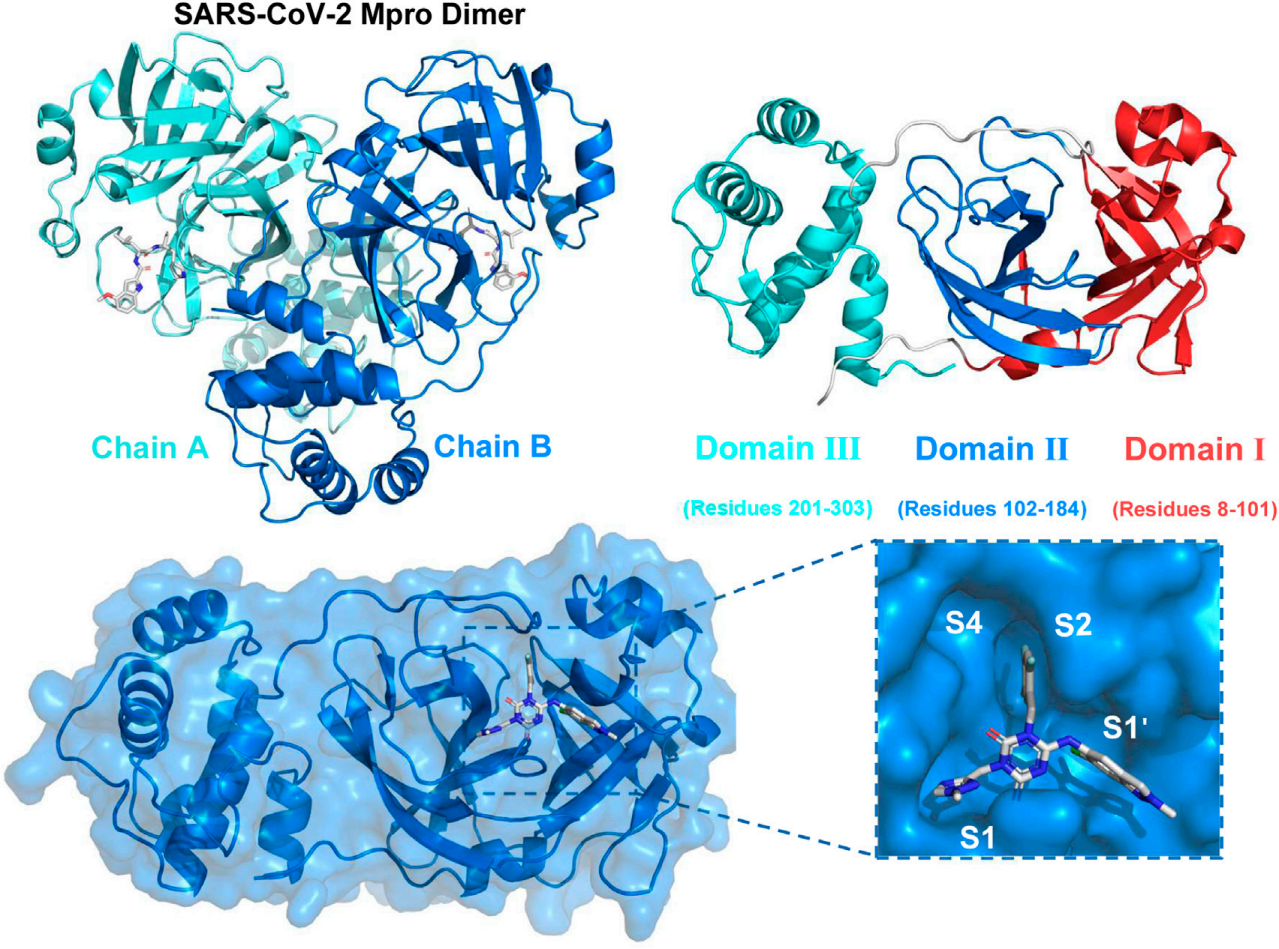
Structure of SARS-CoV-2 Mpro and key active sites S1, S1′, S2 and S4.

SARS-CoV-2 belonged to the Coronaviridae family and was a positive-sense single-stranded RNA virus enclosed in an envelope (Naqvi et al., 2020; Pillaiyar et al., 2020; Wu et al., 2020). The genome of SARS-CoV-2 was approximately 30,000 nucleotides in length and comprised 12 open reading frames that were functional. The pp1a and pp1ab were two proteins that were encoded by genes that overlap with one another. These proteins played a vital role in the process of viral replication and transcription (Mengist et al., 2021). The functional polypeptides were liberated from the polyproteins after substantial proteolytic processing, mostly facilitated by the 33.8 kDa Main protease (Mpro) enzyme, also referred to as 3C-like protease (3CLpro) (Dai et al., 2020; Naqvi et al., 2020). Figure 1 depicted the dimeric structure of 3CLpro comprising two chains (Chain A and Chain B) with each monomer comprising three domains: Domain I (residues 8–101), Domain II (residues 102–184), and Domain III (residues 201–303). The following panel presented the surface representation of the 3CLpro, highlighting the key active sites (S1, S1′, S2, S4), which were crucial for substrate and inhibitor binding (Wu et al., 2023). The 3CLpro enzyme played a crucial role in the replication of the virus by aiding in the production of necessary non-structural proteins (nsps), which enhanced the appeal of this enzyme as a potential target and rendered it appropriate for the creation of wide-ranging anti-coronaviral medications (Iype et al., 2022; Mody et al., 2021).

**FIGURE 2:**
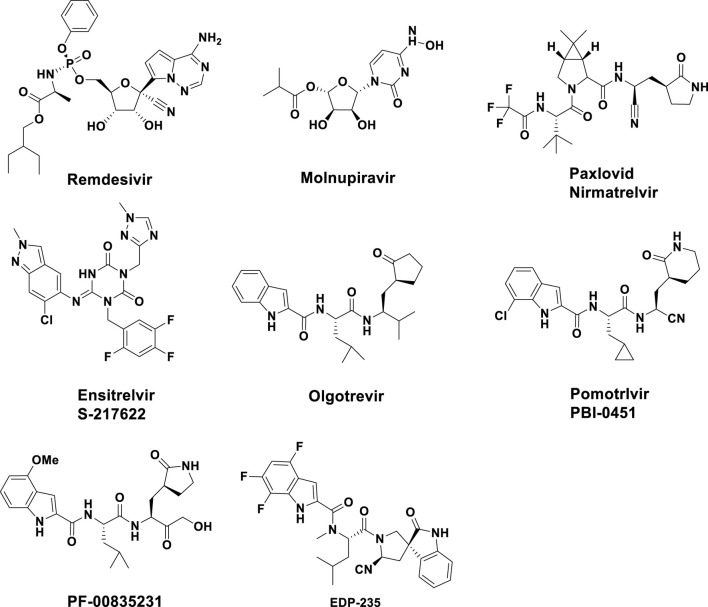
Chemical structures of several Direct-acting antivirals and some known inhibitors of SARS-CoV-2 Mpro.

Several direct-acting antivirals (DAAs) have been given the green light by the Food and Drug Administration (FDA) of the United States of America for the treatment of COVID-19. Remdesivir and Molnupiravir (Jayk Bernal et al., 2022; Wiersinga et al., 2020) were examples of RNA-dependent RNA polymerase (RdRP) inhibitors, while Paxlovid and Ensitrelvir were examples of Mpro inhibitors. However, due to the fact that all four of these medications could only mitigate symptoms and slowed down the progression of the disease, but could not fully suppress the virus, their therapeutic effects were limited. Among them, Remdesivir required intravenous injection, which greatly limited its use (Lai et al., 2020). Monoprevir had the ability to cause mutations in both viral and host cells through a mechanistic process, which raise concerns about its potential to speed up the development of mutant strains and offered hazards to the host (Kabinger et al., 2021). In addition, Paxlovid was considered the most effective and was also the best-selling anti-SARS-CoV-2 drug (Najjar-Debbiny et al., 2023) and Ensitrelvir has demonstrated strong antiviral activity and good pharmacokinetic properties (Mukae et al., 2022). However, long-term use of antiviral drugs increased the risk of drug-resistant strains. There have been many reports of the emergence of drug-resistant strains of Paxlovid and Ensitrelvir (Mukae et al., 2023). Therefore, it remained crucial to continue to develop anti-coronavirus drugs with novel structures and no cross-resistance with existing drugs (Beigel et al., 2020; Wang et al., 2020). In addition to the drugs already on the market, several compounds have also been evaluated as 3CLpro inhibitors, such as Olgotrevir (Mao et al., 2024), Pomotrlvir (Tong et al., 2023), PF-00835231 (Boras et al., 2021), and EDP-235 (Figure 2). However, these compounds were structurally and in binding mode similar to that of Paxlovid and were therefore unlikely to address the resistance issues faced by Paxlovid.

In order to find new structural types of 3CLpro inhibitors to facilitate the solution to the problem of new virus resistance, through the utilization of the recently published crystal structure of 3CLpro in complex with small molecule inhibitors, as well as large-scale virtual screening, evaluation of enzyme inhibitory activity, analysis of binding pattern, and structural stability of the hit compounds with 3CLpro through all-atom molecular dynamics (MD) simulation, we were able to discover new classes of 3CLpro inhibitors. These findings will lay the foundation for the development of new inhibitors.

## 2 Materials and methods

### 2.1 Protein preparation and grid generation

The SARS-CoV-2 3CLpro protein found in the PDB network database (https://www.rcsb.org) with the following PDB ID: 8FY7 and resolution: 1.94 Å was utilized in this research (Ngo et al., 2023). The high-resolution structure of 1.94 Å ensured precise and reliable spatial information, which was crucial for accurate molecular docking and dynamics simulations. The protein was subjected to pre-processing and optimization utilizing the Protein Preparation Wizard available in Schrödinger Release 2023-4. Initially, the spatial configuration was examined for any structural anomalies. Afterwards, hydrogen atoms were incorporated via a hydrogen bond assignment procedure. After eliminating any water or solvent molecules, the protein was optimized using the OPLS4 force field, while keeping all other settings at their default values. The energy-minimized structure was then utilized to create a grid file for ligand docking. The grid box was established by utilizing the native ligand within the 3CLpro as a point of reference, and its dimensions were adjusted to encompass an optimal calculation range during receptor grid setup.

### 2.2 Preparation of ligand library

Topscience database (https://www.tsbiochem.com/service/topscience-database) stands as a leading international chemical database dedicated to supporting the scientific community with a comprehensive collection of active compounds, facilitating drug discovery research. And it is a publicly accessible database of more than 10 million compounds. After an initial screening of over 10 million compounds from the Topscience database using the Lipinski’s rule, the effects of the remaining 5 million compounds on 3CLpro were further examined. The Create Phase Database module, provided by Schrödinger Release 2023-4, was utilized to prepare the ligands by applying specific filters and generating a customized ligand library, which included refining the 3D structures by adding hydrogen atoms, generating stereoisomers, and identifying ionization states. Further ligand refinement was achieved through energy minimization and optimization of the low-energy 3D structure, using the OPLS_2005 force field implemented in PHASE.

### 2.3 Compounds library screening

The stepwise molecular docking process was carried out using the Glide module in Schrödinger to identify the most promising hit compounds. The process began with high throughput virtual screening (HTVS), after which the ligands with the greatest glide scores (top 10%) were chosen for additional analysis. These selected ligands underwent standard precision (SP) docking. The highest-ranking 10% of ligands from the SP docking results were further subjected to extra precision (XP) docking to enhance accuracy and identify the most promising candidates. The Prime module was used to employ the molecular mechanics/generalized born surface area (MM/GBSA) method in order to evaluate the free binding energy between the protein and the docked ligands, which yielded an assessment of the affinities of ligands. The VSGB solvation model and the OPLS4 force field were used for the free binding energy calculations. Additionally, the strain energy of the compounds was calculated using the Strain Energy module. Finally, the most promising compounds were selected based on a combination of their docking scores, calculated binding free energies, and strain energies. The comprehensive evaluation ensured that the top candidates exhibited strong docking interactions, favourable binding affinities, and acceptable strain energies.

### 2.4 ADMET properties

The absorption distribution metabolism excretion toxicity (ADMET) properties of all candidate compounds were analysed by using SwissADME (Daina et al., 2017) (http://www.swissadme. ch/) and ProTox 3.0 (Banerjee et al., 2018; Banerjee et al., 2024) (https://tox.charite.de/protox3/) web facilities. SwissADME was utilized to predict various pharmacokinetic properties such as absorption, distribution, metabolism, and excretion profiles of the compounds. ProTox 3.0 which integrated various models to predict the oral toxicity, hepatotoxicity, cytotoxicity, and potential adverse effects of compounds was employed to assess the toxicity profiles of candidates.

### 2.5 Inhibitory activity from *in vitro* assays

The 44 candidate compounds were purchased from Topscience (Topscience, Shanghai, China). DMSO (Innochem, Beijing, China) was utilized as a solvent for dissolving the compounds, and all solutions were stored at a temperature of −20°C. The screening kit for the SARS-CoV2 3CLpro inhibitor was acquired from the Beyotime Institute of Biotechnology, which was located in Shanghai, China. Fluorescence resonance energy transfer (FRET) method was used to detect the activity of 3CLpro in the novel coronavirus 3CLpro inhibitor screening kit. The 96 wells of black plate were used for the reactions that were carried out. Initially, 91 μL of 3CLpro assay reagent and 5 μL of the test substance were consecutively introduced into each sample well, with DMSO replacing compounds in the model wells. For the control wells, 5 μL of DMSO and 91 μL of assay buffer were introduced. To achieve comprehensive homogenization of the reaction mixture, the 96-well plate was agitated for a duration of 1 min. After that, a rapid addition of 4 μL of the substrate was made to every well, and it was completely mixed. The plate was placed in a dark environment and kept at a temperature of 37°C for a duration of 15–20 min. The measurements of fluorescence were carried out with the assistance of a multifunction enzyme labeling reader (SpectraMax M5, Molecular Devices) that had an excitation wavelength of 325 nm and an emission wavelength of 393 nm. The inhibition rates were determined at single points, and IC_50_ values were plotted using Origin 2024 (n = 6). The inhibition rate was calculated as follows:

$$Inhibition\;rate\;\left(100\%\right)=\frac{{\text{RFU}}_{\text{enzyme}}-{\text{RFU}}_{\text{sample}}}{{\text{RFU}}_{\text{enzyme}}-{\text{RFU}}_{\text{control}}}\times 100\%$$

### 2.6 Molecular dynamics simulations

The GROMACS 2020.7 beta suite was utilized for all-atom MD simulations to analyse the dynamic binding behaviour and stability of protein-candidate compounds complexes. The simulations operated over a 200 ns duration at a temperature of 298.15 K by utilizing the Amber ff14SB force field (Maier et al., 2015). Parameters for the ligands were generated by using the general amber force field (GAFF) parameters (Wang et al., 2004) the fitted charges were calculated at the B3LYP/cc-pVDZ level by using Gaussian 16 (Frisch et al., 2016), Multiwfn (Lu and Chen, 2012) and Sobtop (Lu, 2022) software. For each system, solvation was performed using a 10 Å cubic box filled with TIP3P water molecules. The genion module in GROMACS was employed to introduce the necessary counter ions (Na^+^ and Cl^−^) to neutralize the system. The energy minimization process was conducted with periodic boundary conditions and utilized the steepest descent algorithm over 5,000 steps. Electrostatic interactions were computed using the Particle Mesh Ewald (PME) method. Using the V-rescale temperature coupling approach under the NVT ensemble, the system temperature was progressively raised from 0 K to 298.15 K. Afterwards, the NPT ensemble’s Parrinello-Rahman barostat was used to keep the system pressure at 1 atm. Finally, each system was subjected to a 200 ns molecular dynamics simulation to ensure that the systems reached equilibrium and stability.

### 2.7 DFT calculations

The DFT is used to ascertain the electron’s density and energy properties. Gaussian 16 program was employed to perform analysis of compounds with visualization conducted using GaussView 6.0. The structural coordinates of compounds were optimized at the B3LYP/6–311++G (d,p) level of theory without imposing any symmetry constraints. The molecules have one multiplicity and zero charges. The electrostatic surface potential and HOMO-LUMO energy levels of compounds were derived from the optimized geometry utilizing Multiwfn.

## 3 Results and discussion

**FIGURE 3:**
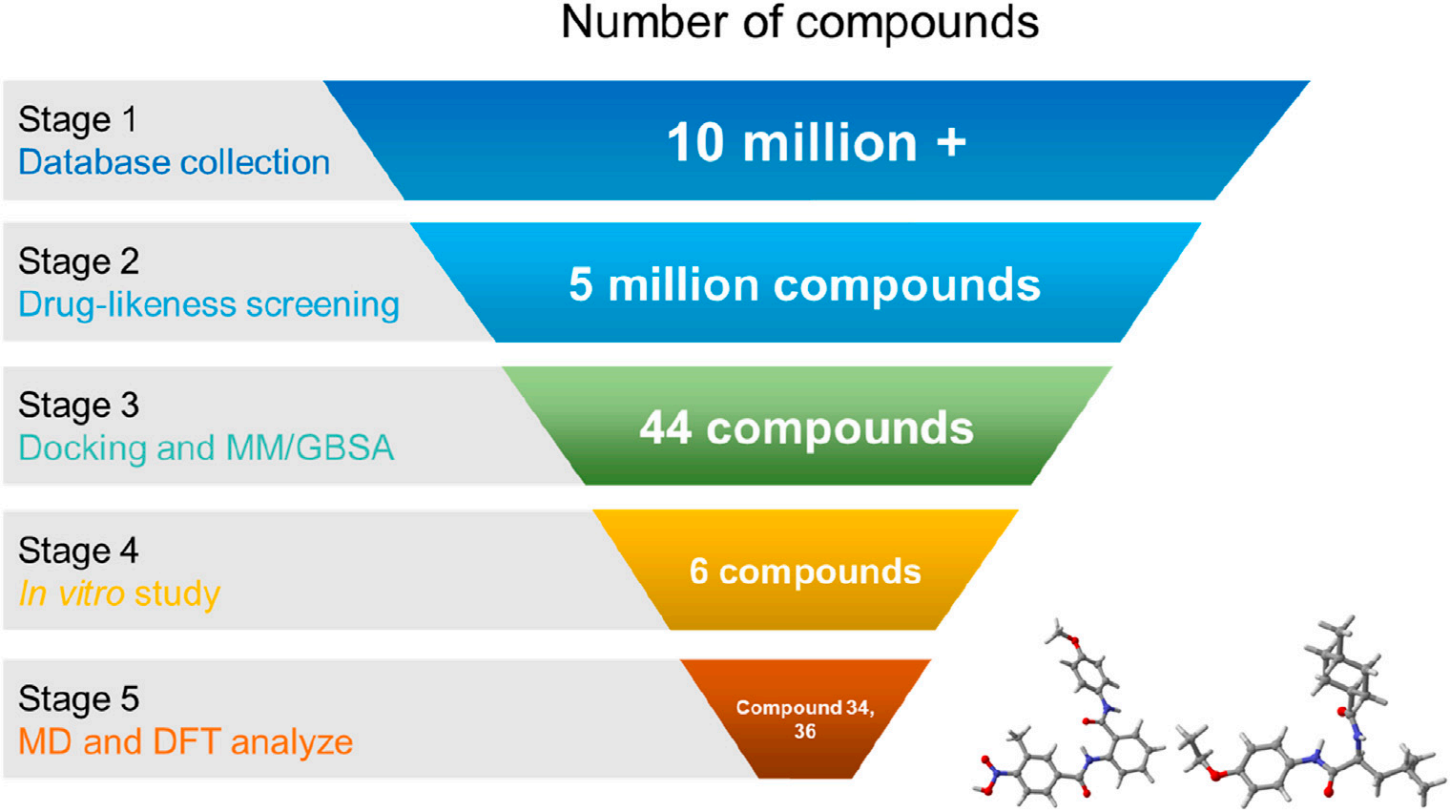
Structure-based virtual screening scheme of potent 3CLpro inhibitor.

The workflow illustrated in Figure 3 detailed a methodical approach for identifying potent 3CLpro inhibitors. Initial database collection encompassed over 10 million compounds, which were subsequently filtered through drug-likeness screening, yielding approximately 5 million. These were subjected to docking studies calculation of MM/GBSA, calculation of strain energy, and evaluation of ADMET profiles, narrowing the selection to 44 compounds. Further *in vitro* evaluation identified six promising candidates. The final phase involved comprehensive binding, molecular dynamics and density functional theory (DFT) analyses, with a particular focus on compounds 34 and 36, to elucidate their interactions with the 3CLpro enzyme. The rigorous process provided critical insights and potential scaffolds for the development of effective antiviral therapeutics.

### 3.1 Molecular docking and ADMET assessment

Molecular docking is a crucial and essential technology that provides a valuable alternative to *in vitro* screening in the field of drug discovery (Stanzione et al., 2021). Schrödinger’s software, particularly the Glide module, was chosen for molecular docking due to its high accuracy, comprehensive suite of integrated tools, user-friendly interface, and advanced scoring functions. Before the molecular docking of 3CLpro, docking validation was conducted to ensure the reliability of the process, with the results available in the (Supplementary Figure S1). The molecular docking of 3CLpro was performed on 5 million compounds obtained by preliminary screening using Lipinski’s rule from the Topscience database. These candidate compounds were identified through a rigorous selection process. The initial screening considered the XP GScore, a metric that predicts the binding affinity of compounds to the target protein. Compounds with the most favourable XP GScore were then subjected to further analysis using MM/GBSA binding free energy calculations to provide a more accurate estimation of their binding affinities. Strain energy was also evaluated to ensure that the binding of these compounds did not induce significant conformational changes in the target protein, which could affect the stability of the protein-ligand complex (Supplementary Table S1). Lower strain energy was generally favorable as it suggested less conformational distortion upon binding.

**FIGURE 4:**
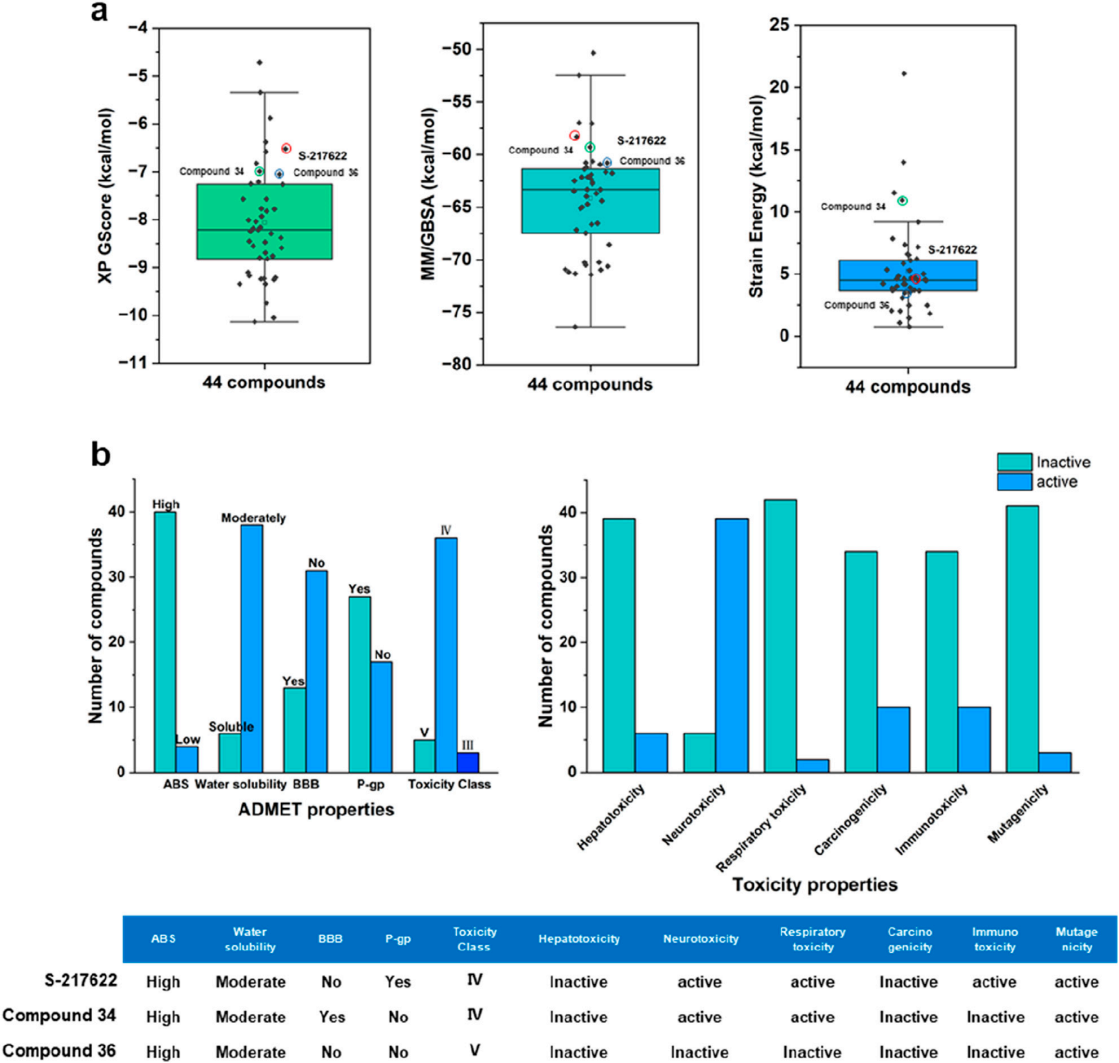
**(A)** Box plots showing the distribution of XP GScore, MM/GBSA binding free energy, and strain energy for the top 44 compounds, with S-217622 highlighted. **(B)** ADMET properties and toxicity profiles of the top 44 compounds and S-217622.

Figure 4 provided a visual representation of key metrics used in the selection process. Figure 4A showed box plots of the distribution of XP GScore, MM/GBSA binding free energy, and strain energy for the top 44 compounds, with S-217622 highlighted. The compound S-217622 was chosen as a reference due to its noncovalent, nonpeptidic nature, broad-spectrum antiviral activity against SARS-CoV-2 variants, favorable pharmacokinetic profile and efficacy in animal models. These box plots illustrated that most of the top 44 compounds had more favorable XP GScore and MM/GBSA binding free energy compared to S-217622, indicating stronger binding affinities and stable binding. The XP GScore ranged from −10.13 to −4.72 kcal/mol among the compounds, with compound 15 having the lowest score (−10.13 kcal/mol), suggesting the strongest binding affinity. And S-217622 had a relatively moderate XP GScore of −6.58 kcal/mol. The ∆G bind values ranged from −76.38 kcal/mol to −50.32 kcal/mol, with compound 11 showing the most stable binding (−76.38 kcal/mol). S-217622 had a ∆G bind of −57.03 kcal/mol, which was relatively moderate compared to other compounds. The strain energy for most compounds was less than 10 kcal/mol, indicating minimal conformational changes. Besides, S-217622 had a strain energy of 5.300 kcal/mol, which was within an acceptable range but not the lowest (Supplementary Table S1).

The evaluation of properties is pivotal in pinpointing molecules that hold the greatest promise as effective drugs for particular diseases during the drug design and development phases (Zhou et al., 2016). These evaluations focus on determining the compounds’ capabilities for absorption, their bodily distribution and mobility, their metabolism and excretion potential, and the assessment of any related toxicological implications (Jana et al., 2018). In addition to the inhibitory efficacy, a comprehensive evaluation of the ADMET properties of the lead compounds was conducted to ensure their potential as drug candidates (Supplementary Tables S2, S3). Figure 4B summarized the ADMET properties and toxicity profiles for top 44 compounds compared to S-217622. Most compounds exhibited high absorption and moderate water solubility, with a balanced distribution regarding BBB penetration and P-gp interaction. The majority fell into toxicity class IV. In terms of toxicity, while many compounds were inactive for hepatotoxicity, respiratory toxicity, and carcinogenicity, a significant number showed activity for neurotoxicity, immunotoxicity, and mutagenicity. S-217622 displayed high absorption, moderate water solubility, no BBB penetration, P-gp interaction, and fell into toxicity class IV, with specific activities in neurotoxicity, respiratory toxicity, immunotoxicity, and mutagenicity. These findings highlighted the strengths and potential safety concerns of the top 44 compounds as 3CLpro inhibitors. After comprehensive analysis of multiple parameters, we selected and purchased 44 compounds for wet screening.

Although this study successfully identified potential SARS-CoV-2 3CLpro inhibitors using traditional computer-aided drug design (CADD) methods, the role of artificial intelligence (AI) in accelerating the drug discovery process has gained significant attention in recent years. AI technologies can rapidly process large-scale data, automate compound screening, and predict compound activity, selectivity, and ADMET properties, offering a time- and resource-efficient alternative to traditional methods. Future studies could explore how AI techniques can be integrated with CADD methods, particularly to expedite the discovery of SARS-CoV-2 3CLpro inhibitors, enhancing the efficiency of identifying potential drug candidates and reducing the cost of experimental screening.

### 3.2 Inhibitory activity from *in vitro* assays

The top 44 compounds were evaluated and experimentally analysed to confirm their inhibitory action against 3CLpro. The inhibitory activity of 3CLpro was assessed using the FRET technique. There was no difference between the sequence of amino acids found in 3CLpro and that of the natural new coronavirus 3CLpro. The detection mechanism involves linking the fluorescent donor (MCA) and fluorescent receptor (Dnp) to both ends of the natural substrate of the 3CLpro protease. Specifically, this substrate is MCA-avlqsgfr-lys (Dnp)-Lys-NH2. After 3CLpro cuts the substrate, fluorescence of MCA can be detected. The initial screens were conducted with inhibitor doses of 50 μM (Supplementary Table S4). Among the tested compounds, compound 1 exhibited a notable inhibition percentage of 85.4%, closely followed by compounds 43 (84.8%), 5 (84.0%), 36 (81.9%), and 34 (78.3%). Compound 10 also demonstrated significant inhibitory activity at 78.0%. Notably, the reference compound S-217622 displayed the highest inhibition at 99.2%, serving as a benchmark for evaluating the efficacy of the tested compounds. Overall, the screening identified several potent inhibitors of 3CLpro, particularly compounds 1, 5, 10, 34, 36, and 43 which demonstrated inhibition percentages above 70%. These results highlighted the potential of these compounds as lead candidates for further optimization and development of effective antiviral therapies against SARS-CoV-2.

**FIGURE 5:**
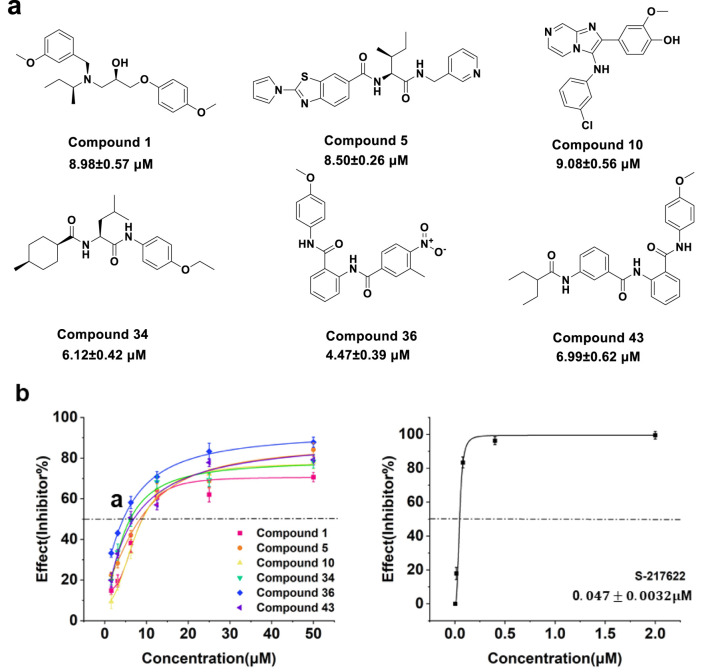
**(A)** Structure and IC_50_ value of compounds 1, 5, 10, 34, 36 and 43. **(B)** Concentration response curve of compounds 1, 5, 10, 34, 36, 43 and S-217622 against 3CLpro activity.

To gain further insights into the potency of these compounds, we conducted concentration-response assays to determine their IC_50_ values. Figure 5A illustrated the structure and IC_50_ values for selected compounds against 3CLpro activity. Figure 5B presented the inhibition curves for compounds 1, 5, 10, 34, 36, 43, and the reference compound S-217622, showing their effectiveness across a range of concentrations. Among these, compound 36 demonstrated the highest potency with an IC_50_ value of 4.47 ± 0.39 μ M, followed by compound 34 (6.12 ± 0.42 μ M). The inhibition curve for the reference compound S-217622 displayed a significantly lower IC_50_ value of 0.047 ± 0.0032 μ M, which was consistent with the activity data reported in the literature. These results suggested that while the tested compounds exhibit promising inhibitory effects on SARS-CoV-2 3CLpro, particularly compounds 34 and 36, they are less potent than the reference inhibitor S-217622. Further optimization and structural modifications may enhance their efficacy, providing a foundation for developing effective antiviral therapies.

### 3.3 Molecular dynamics simulation

The all-atom MD simulations further examined the effects of explicit solvent molecules on the protein, focusing on fluctuations and conformational adjustments. Additionally, the simulations enabled the collection of time-averaged attributes of the complex system over different timescales (Wichapong et al., 2013). From the evaluation of comprehensive calculation and pharmacological results, compounds 34 and 36 were identified as outstanding candidates. Consequently, ligand-free 3CLpro (apo-3CLpro), and the docked complexes of 3CLpro with compounds 34 and 36 were selected for all-atom MD simulation studies, with an aim to evaluate the stability and dynamic behaviour of compounds 34 and 36 when complexed with the 3CLpro, providing deeper insights into their potential as effective inhibitors. To ensure the robustness and accuracy of the results, three independent MD simulations were conducted for each system. The RMSD values across all three trajectories were nearly identical, confirming the consistency and reliability of the simulations.

#### 3.3.1 Structural deviations and compactness

**FIGURE 6:**
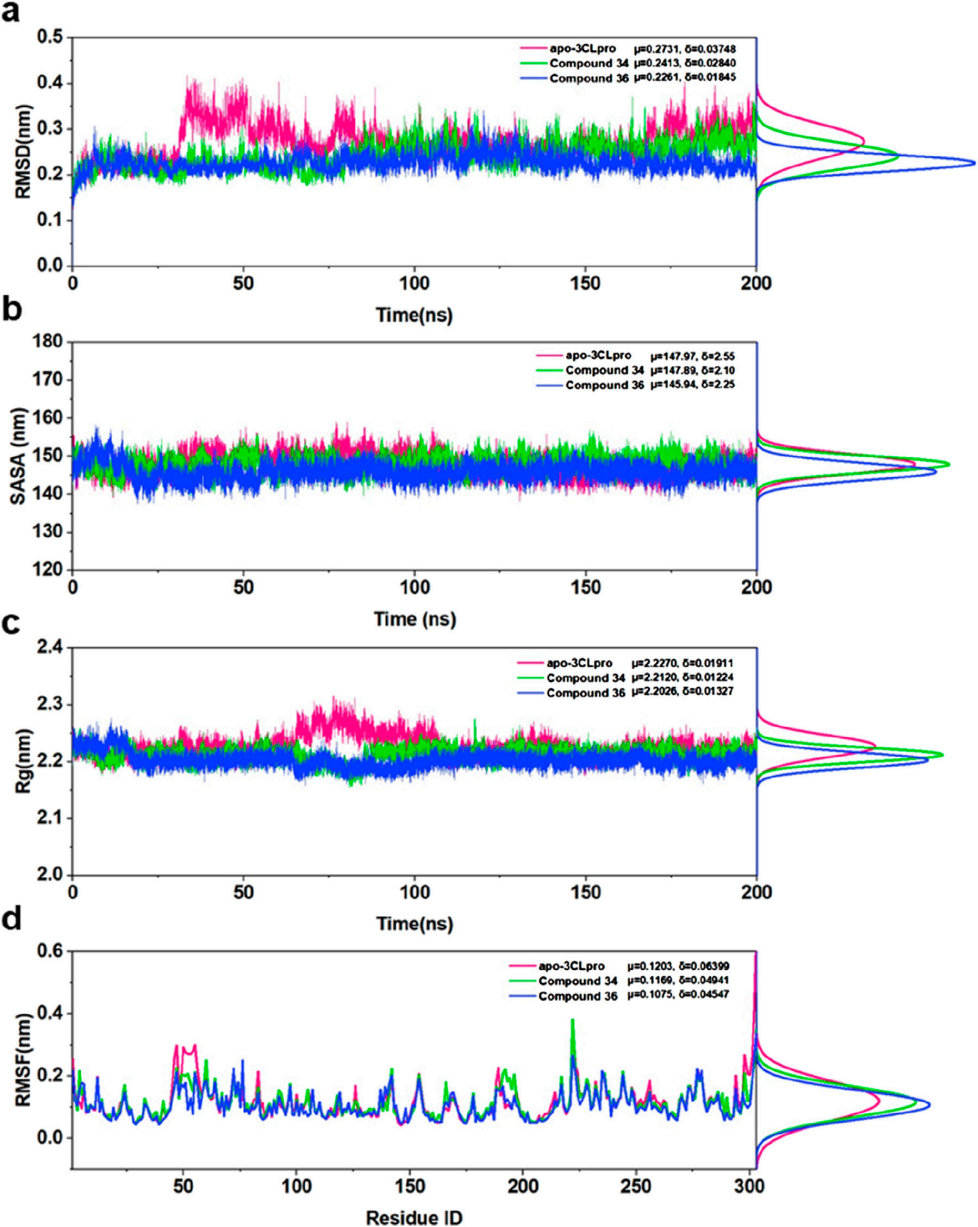
**(A)** RMSD for apo-3CLpro, compound 34 and 36 complexes over 200 ns along with their respective distributions. **(B)** SASA for apo-3CLpro, compound 34 and 36 complexes over 200 ns along with their respective distributions. **(C)** Rg for apo-3CLpro, compound 34 and 36 complexes over 200 ns along with their respective distributions. **(D)** RMSF for apo-3CLpro, compound 34 and 36 complexes over last 50 ns, along with their respective distributions.

The root means square deviation (RMSD) analysis (Figure 6A) indicated that the apo-3CLpro system exhibited higher fluctuation levels over the 200 ns simulation period compared to the complexes with compounds 34 and 36. The mean RMSD values for apo-3CLpro, compound 34 and 36 complexes were 0.2731 nm, 0.2431 nm, and 0.2281 nm, respectively, with corresponding standard deviations of 0.03748 nm, 0.02840 nm, and 0.01845 nm. The lower mean RMSD and reduced variability in the complexes suggested enhanced structural stability upon binding with compounds 34 and 36. The solvent accessible surface area (SASA) results (Figure 6B) further supported this observation, as the apo-3CLpro system showed greater variability in SASA compared to compounds 34 and 36 complexes. The mean SASA values for apo-3CLpro, compounds 34 and 36 complexes were 147.97 nm^2^, 147.89 nm^2^, and 145.94 nm^2^, respectively, with standard deviations of 6.25 nm^2^, 6.20 nm^2^, and 6.25 nm^2^. The consistent SASA values for the compounds 34 and 36 complexes implied a more compact structure with reduced solvent exposure, indicative of a stabilized protein conformation. Analysis of the radius of gyration (Rg) (Figure 6C) showed that the overall compactness of the protein structure was better maintained in the presence of compounds 34 and 36 complexes. The mean Rg values for apo-3CLpro, compounds 34 and 36 complexes were 2.2270 nm, 2.2210 nm, and 2.2206 nm, respectively, with standard deviations of 0.01911 nm, 0.01224 nm, and 0.01327 nm. The lower mean Rg values for the complexes, as compared to the apo form, suggested that these compounds 34 and 36 promote a more stable and compact protein structure.

Finally, the root means square fluctuation (RMSF) profiles (Figure 6D) demonstrated that the fluctuations of individual residues were significantly reduced in compounds 34 and 36 complexes. The mean RMSF values for apo-3CLpro, compounds 34 and 36 complexes were 0.1203 nm, 0.1169 nm, and 0.1075 nm, respectively, with standard deviations of 0.06399 nm, 0.04841 nm, and 0.04547 nm. This reduction in residue-wise fluctuations highlighted the stabilizing effect of compounds 34 and 36 on the dynamic regions of the protein. In conclusion, the binding of compounds 34 and 36 to the 3CLpro resulted in enhanced structural stability and reduced conformational flexibility, as evidenced by the lower mean RMSD, SASA, Rg, and RMSF values, along with reduced variability. These findings underscored the potential of compounds 34 and 36 as effective inhibitors of 3CLpro, providing a strong basis for further experimental validation and development as therapeutic agents.

#### 3.3.2 Principal component analysis

**FIGURE 7:**
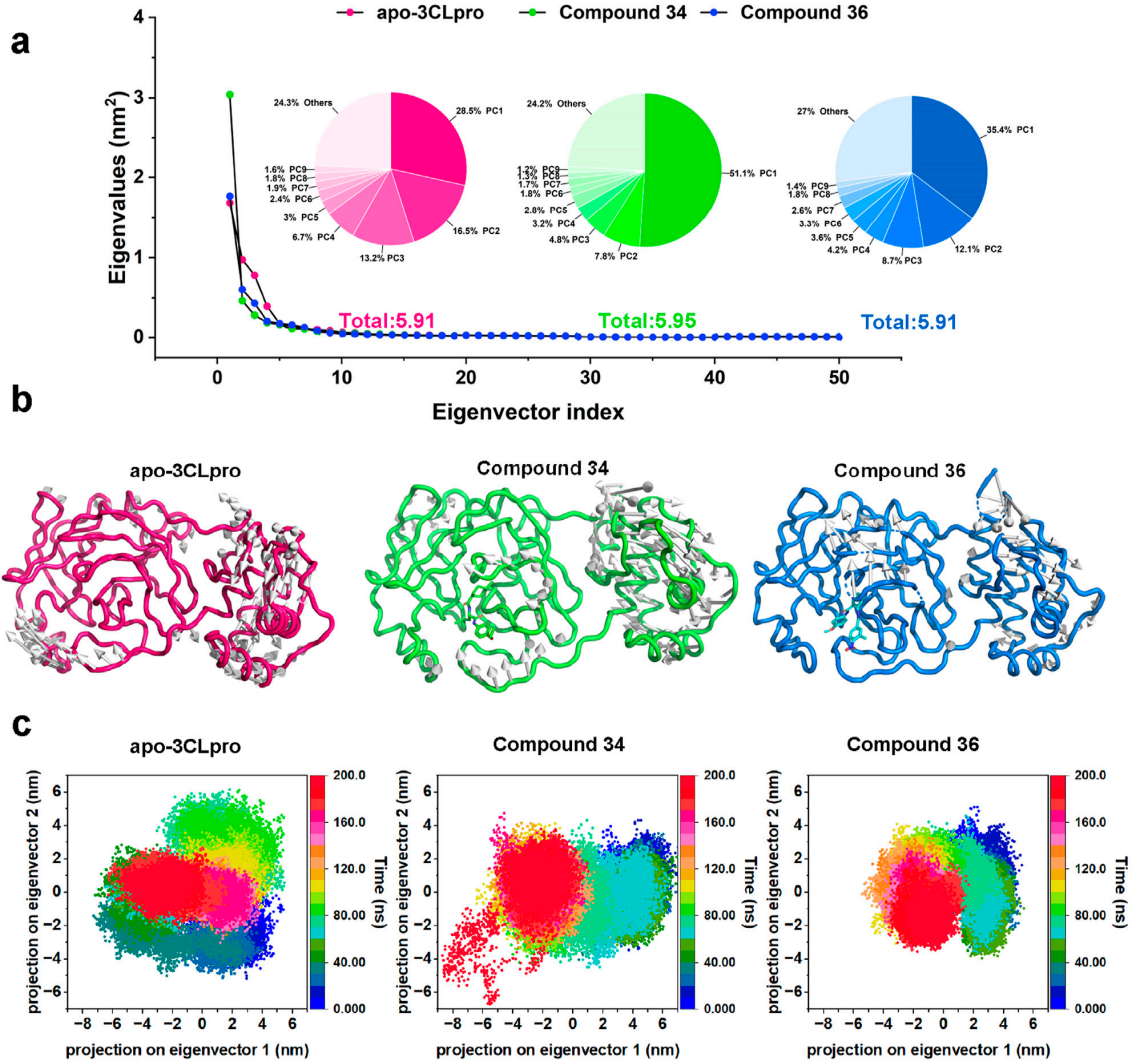
**(A)** Dotted line plots representing the first 50 eigenvectors and corresponding eigenvalues of apo-3CLpro, compound 34 and 36 complexes. **(B)** Projection of the first principal component onto the protein structure to visualize the motion direction of each residue. **(C)** Representation of the 2D projections of apo-3CLpro, compound 34 and 36 complexes conformational changes during the simulation.

Subsequently, to further substantiate these findings and provide a comprehensive understanding of the conformational dynamics, principal component analysis (PCA) was employed by constructing a covariance matrix for all Cα atoms of 3CLpro. PCA was a powerful technique that helps in identifying the most significant modes of motion within the protein-ligand complexes, and the eigenvectors and eigenvalues were used to describe the motion mode and the corresponding motion intensity, respectively. The eigenvalue plots (Figure 7A) illustrated the distribution of the first 50 eigenvectors for the apo-3CLpro, compounds 34 and 36 complexes. Notably, the apo-3CLpro system and compound 34 complex exhibited higher eigenvalues of 5.91 nm^2^ and 5.95 nm^2^ compared to the compound 36 complex with eigenvalues of 4.99 nm^2^, indicating greater conformational flexibility. In contrast, the complexes with compound 36 showed significantly reduced eigenvalues for these components, suggesting that the binding of these compounds imposed a more rigid and stable conformational state on the protein. However, for the motion described by the first principal component (PC1), the compounds 34 and 36 complexes had higher eigenvalues than the apo-3CLpro system, with 51.1% and 35.4% of the overall motion, respectively. Moreover, for the other eigenvectors, the eigenvalues corresponding to the compounds 34 and 36 complexes decreased rapidly and were all lower than those of the apo-3CLpro system, implying that the complex system possessed a more concentrated motion mode compared to apo-3CLpro.

The projection of the first principal component onto the protein structure (Figure 7B) provided a visualization of the dominant motions within the systems. The apo-3CLpro displayed more extensive motions, particularly in certain flexible regions. However, these motions were markedly reduced in the presence of compounds 34 and 36, indicating that these compounds conferred substantial stabilization to the protein structure. The stabilization effect was particularly critical for regions essential for the protein’s function, where reduced flexibility may enhance the binding efficiency and overall inhibitory potential of the compounds. The two-dimensional projections of the conformational changes during the simulation (Figure 7C) further elucidated the impact of compounds 34 and 36 on the dynamics of 3CLpro. The apo-3CLpro system demonstrated a broader range of conformational states, while the presence of compounds 34 and 36 resulted in a more constrained conformational space. The confinement in the conformational space reflected the stabilizing effect of the compounds, leading to fewer structural fluctuations and more defined conformations. In conclusion, the PCA and conformational projection analyses revealed that compounds 34 and 36 significantly enhance the stability of the 3CLpro, reducing its conformational variability and internal motions. These findings, consistent with the RMSD, SASA, Rg, and RMSF analyses, underscored the potential of compounds 34 and 36 as potent 3CLpro inhibitors.

#### 3.3.3 Free energy landscape analysis and dynamic cross-correlation matrix analysis

**FIGURE 8:**
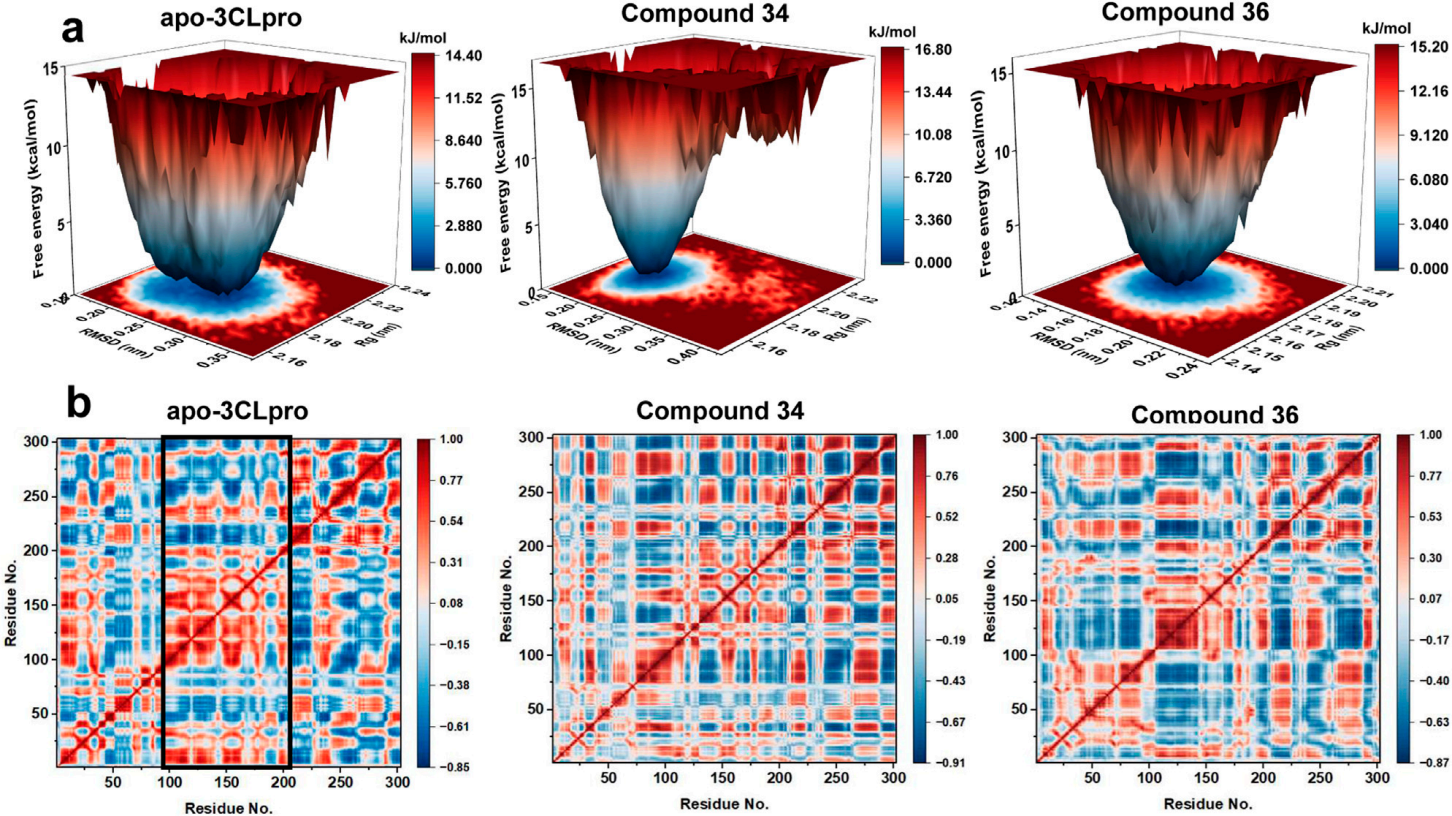
**(A)** Free energy landscapes for apo-3CLpro, compound 34 and 36 systems. **(B)** Dynamic cross-correlation map of the Cα atoms of apo-3CLpro, compound 34 and 36 systems.

Free energy landscapes (FEL) were crucial for understanding the energetic stability and conformational states of the protein-ligand complexes, while dynamic cross-correlation matrix (DCCM) was essential for examining the correlated motions between residues and identifying key interaction networks. Therefore, to provide deeper insights into the stability and interactions within the complexes, FEL and DCCM analyses were performed. The free energy landscapes (Figure 8A) provided insights into the stability and conformational states of the systems. The apo-3CLpro exhibited a broader and deeper energy basin, indicating a higher degree of conformational flexibility and multiple stable states. In contrast, the energy landscapes for the complexes with compounds 34 and 36 were more confined and exhibit shallower basins, suggesting a more restricted conformational space and enhanced stability. The reduction in the conformational diversity implied that the binding of compounds 34 and 36 stabilizes the protein, reducing its free energy and favouring a more stable structure.

The DCCM (Figure 8B) further elucidated the impact of compounds 34 and 36 on the internal dynamics of the 3CLpro. In the apo form, there were extensive positive and negative correlations between different residues, indicating a high level of internal motion and flexibility. However, upon binding with compounds 34 and 36, these correlations were significantly reduced, particularly in key functional regions of the protein (Residues 100–200). This reduction in correlated motions suggested that the compounds conferred rigidity to the protein, thereby stabilizing its structure and potentially enhancing its inhibitory effectiveness. In conclusion, the FEL and DCCM analyses demonstrated that compounds 34 and 36 significantly stabilized the 3CLpro protein, reducing its conformational flexibility and internal dynamics. These findings aligned with the RMSD, SASA, Rg, RMSF, and PCA analyses, further supporting the potential of compounds 34 and 36 as potent 3CLpro inhibitors. The enhanced stability and reduced flexibility upon binding indicated promising therapeutic candidates, meriting further experimental validation and development.

#### 3.3.4 Protein-ligand interactions

**FIGURE 9:**
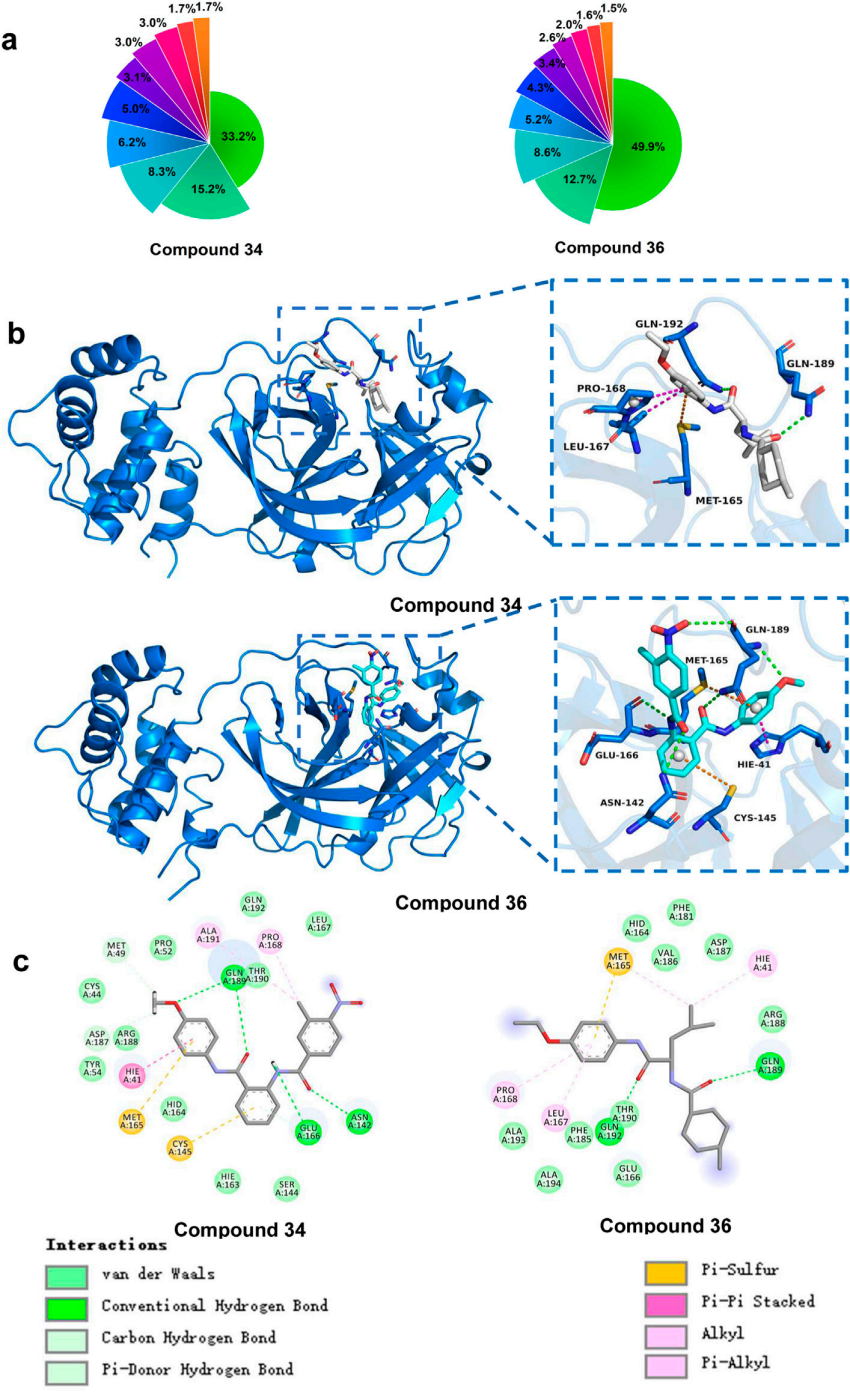
**(A)** Percentage of the top 10 conformational clusters to the total conformational clusters. **(B)** Protein-ligand interactions of compound 34 and 36 with 3CLpro (3D). **(C)** Protein-ligand interactions of compound 34 and 36 with 3CLpro (2D).

Understanding and analysing the interactions between proteins and their ligands was essential for developing new and effective treatments. To obtain the most representative conformations, clustering analysis was performed on the trajectory from the final 50 ns of the stable phase. The Figure 9A presented the distribution of the top 10 conformational clusters which constituted the highest proportion of conformations for compounds 34 and 36 complexes. It demonstrated that both compounds 34 and 36 complexes had dominant conformations that were significantly prevalent, suggesting these were the most stable and potentially most relevant for interactions with 3CLpro. In particular, compound 36 complex showed a very high dominance of a single conformation (49.9%), suggesting a strong preference for a specific binding mode. These results were crucial for understanding the stability and binding interactions of these compounds with 3CLpro, potentially guiding further drug development efforts.

Building on this analysis, the most representative conformations were selected from the highest proportion of conformations cluster. Compounds 34 and 36 exhibited strong and stable interactions with critical active site residues of 3CLpro presented in Figure 9B. These interactions included van der Waals (vdW) forces, conventional hydrogen bonds, carbon hydrogen bonds, Pi-donor hydrogen bonds, Pi-sulfur interactions, Pi-Pi stacking, and alkyl interactions. For compound 34, the interaction diagram revealed multiple hydrogen bonds with residues such as Gln189, Thr190, and Gln192, which played significant roles in stabilizing the ligand within the active site. Additionally, vdW interactions with residues like Ala191 and Met165 further contributed to the ligand’s binding affinity and stability. Unlike S-217622, which primarily occupied the S1, S1′ and S2 sites, compound 34 engaged the S4 and S1′ active sites, demonstrating a unique binding mode.

Similarly, compound 36 formed strong hydrogen bonds with key residues, including Gln189. The presence of Pi-Pi stacking interactions with Hie41 and Pi-sulfur interactions with Met49 enhanced the binding affinity, ensuring a stable protein-ligand complex. Compound 36 was particularly notable for occupying the S1, S2, and S4 sites, which differed from the binding pattern of S-217622, suggesting a novel interaction profile. The vdW interactions with residues such as Met165 and Glu166 further supported the ligand’s stable binding. The overall binding interaction profiles of compounds 34 and 36 with 3CLpro suggested that these compounds fitted well within the active site, forming stable and diverse interactions. These strong and varied interactions were likely to contribute to the high inhibitory potential of the compounds against the 3CLpro.

In terms of novelty, both compounds 34 and 36 exhibited distinctive structural features and binding behaviors when compared to known inhibitors such as S-217622. Their distinct binding modes occupying previously unexplored sites like S4 and forming non-conventional interactions indicated that these molecules were exploring new chemical space. This suggested opportunities for further optimization of their chemical structures, potentially improving their binding affinity, selectivity, and pharmacokinetic properties. Moreover, there remained significant chemical space to explore. First, the diverse binding interactions these compounds exhibited with the active site residues suggested that targeted modifications could have further enhanced their binding affinity and selectivity. For instance, structural modifications of their side chains could have been explored to improve hydrophobicity, polarity, or electronic effects, which might have resulted in better drug-like properties. Second, although compounds 34 and 36 showed novel binding modes, their chemical scaffolds could have been further optimized to explore broader chemical space. Alterations to the core structure could have yielded derivatives with improved potency and efficacy against 3CLpro. Future studies that combined AI methodologies and experimental validation could have further optimized these compounds, unlocking their full potential as 3CLpro inhibitors.

### 3.4 DFT analysis

**FIGURE 10:**
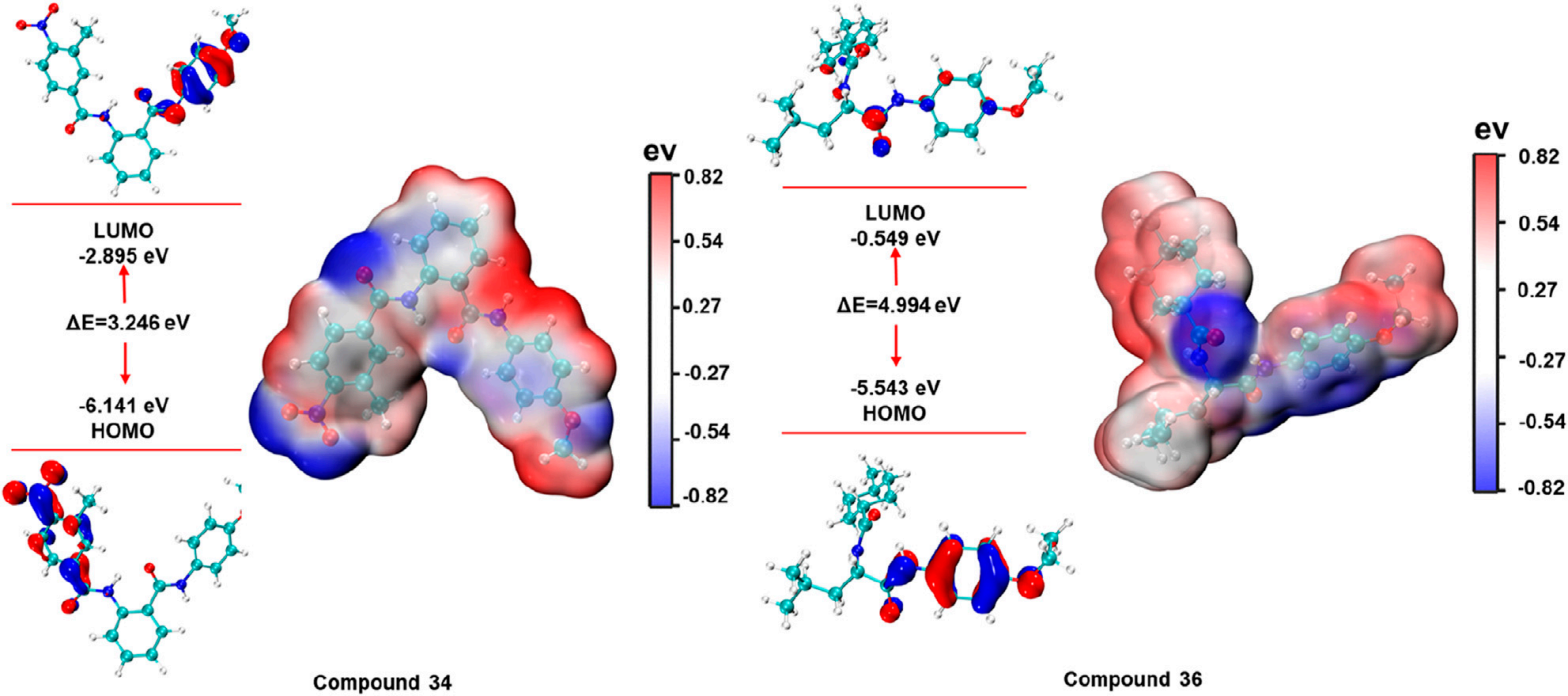
HOMO-LUMO energy plot and electron density surface map of compounds 34 and 36.

The energies of the HOMO and LUMO boundary orbitals play an essential part in the field of quantum chemistry as they affect the behaviour of a molecule reaction to other compounds (Guerroudj et al., 2021). Moreover, the boundary orbit makes it easy to characterize the chemical reactivity as well as the kinetic stability of analysed molecule (Hao et al., 2020). The electronic properties and molecular interactions of compounds 34 and 36 were thoroughly examined through frontier molecular orbital analysis and molecular electrostatic potential surfaces, as depicted in Figure 10. Compound 34 exhibited a HOMO energy of −6.141 eV and a LUMO energy of −2.895 eV, resulting in a gap of 3.246 eV. In contrast, compound 36 had a HOMO energy of −5.543 eV and a LUMO energy of −0.549 eV, producing a much larger gap of 4.994 eV. The smaller HOMO-LUMO gap in compound 34 suggested higher reactivity, which could correlate with a greater ability to participate in charge transfer interactions or other electron-related mechanisms with the biological target. Meanwhile, the larger gap in compound 36 indicated greater stability and potentially lower reactivity.

The molecular electrostatic potential maps provide further insights into the charge distribution across the molecular surface. Both compounds exhibit distinct patterns of positive and negative regions, marked by red (negative) and blue (positive) zones. For compound 34, the molecular electrostatic potential map indicated a significant region of negative potential localized near electronegative atoms, which suggested potential for forming strong hydrogen bonds or electrostatic interactions with positively charged residues in the protein binding site. On the other hand, compound 36 showed a more balanced distribution of positive and negative potential, with the central part of the molecule bearing considerable electron density. These analyses provided valuable insights into the reactivity, stability, and potential binding affinities of the compounds with their respective biological targets.

## 4 Conclusion

In this study, the novel antiviral inhibitors against SARS-CoV-2 3CLpro were identified by using *in silico* and *in vitro* approaches. Initially, the molecular docking was performed on 5 million compounds obtained by preliminary screening of Lipinski’s rule from the Topscience database. After the HTVS, SP, XP docking, calculation of MM/GBSA, calculation of strain energy and evaluation of ADMET profiles, 44 compounds were chosen to test the inhibitory activity of targeting 3CLpro. Secondly, *in vitro* pharmacological assays the inhibitory efficacy of compounds 34 and 36 against SARS-CoV-2 3CLpro, with IC_50_ values of 6.12 ± 0.42 μM and 4.47 ± 0.39 μM, respectively. Subsequently, the results of all-atom MD simulations revealed that compounds 34 and 36 significantly enhanced the structural stability of the 3CLpro, as evidenced by lower RMSD, SASA, Rg, and RMSF values, along with reduced variability compared to the apo-3CLpro system. The analysis of PCA indicated that compounds 34 and 36 reduced the conformational flexibility and internal motions of 3CLpro, further stabilizing its structure. The analysis of FEL and DCCM showed that compounds 34 and 36 restricted the conformational space and reduced correlated motions within the protein, and leading to a more stable and less dynamic structure. Furthermore, protein-ligand interaction studies highlighted that there were strong and diverse interactions formed between compounds 34 and 36 with key active site residues of 3CLpro, including hydrogen bonds, vdW forces, Pi-Pi stacking, and Pi-sulfur interactions. In addition, unlike known inhibitors, compound 34 and 36 were unique binding modes with SARS-CoV-2 3CLpro, compound 34 occupied the S4 and S1′ active sites, while compound 36 engaged the S1, S2, and S4 sites. Compound 34s smaller HOMO-LUMO gap and localized negative potential suggest higher reactivity and binding affinity. Besides, compound 36 with a larger HOMO-LUMO gap and more balanced electrostatic potential, demonstrates greater stability.

In future research, deep learning techniques could be applied to MD simulation data to systematically compare the conformational dynamics between inhibitor-bound and apo-protein systems. This approach could provide deeper insights into key structural regions that influence inhibitor functionality, complementing the traditional methodologies employed in this study. These findings suggested that compounds 34 and 36 could have served as promising lead compounds for the development of novel SARS-CoV-2 3CLpro inhibitors. Their distinct interactions, favourable pharmacological profiles and robust MD simulation data, underscored their potential as promising therapeutic candidates for the treatment of COVID-19.

## Data Availability

The raw data supporting the conclusions of this article will be made available by the authors, without undue reservation.
