# Supplementary material for: Identification of novel SARS-CoV-2 3CLpro inhibitors by molecular docking, in vitro assays, molecular dynamics simulations and DFT analyses
Source: Front Pharmacol. 2024 Oct 30;15:1494953. doi: 10.3389/fphar.2024.1494953 (PMC11557435; doi:10.3389/fphar.2024.1494953)
Supplement: Supplementary file 1 [file DataSheet1.docx]

# *Supplementary Material*

**Identification of novel SARS-CoV-2 3CLpro inhibitors by molecular docking, *in vitro* assays, molecular dynamics simulations and DFT analyses.**

Keli Zong ^a b †^, Chaochun Wei ^a †^, Wei Li ^b†^, Jiajun Ruan ^b^ Susu Zhang ^b^, Jingjing Li ^a^, Xiaojing Liu ^b^, Xu Zhao ^c^ Ruiyuan Cao* ^b^ Hong Yan ^a^ * and Xingzhou Li ^b^ *

*^a^College of Chemistry and Life Science, Beijing University of Technology, Beijing 100124, P. R. China*

*^b^Beijing Institute of Pharmacology and Toxicology, 27 Taiping Road, Beijing 100850, China*

*^c^Department of Hepatology, Fifth Medical Center of Chinese PLA General Hospital, 100 West Fourth Ring Road, Beijing100071, China*

^†^These authors contribute equally; * Correspondence author.

* Correspondence e-mail: [caoruiyuan@bmi.ac.cn](mailto:caoruiyuan@bmi.ac.cn), hongyan@bjut.edu.cn, and lixz@bmi.ac.cn

## Molecular Docking Validation

To confirm the reliability and accuracy of our docking approach, we first attempted to re-dock the original ligand into its 3CLpro binding site using the Glide module. The docking was performed with XP settings, allowing flexibility of the ligand as per default parameters, followed by a post-docking minimization step. The RMSD between the highest-scoring pose (XP GScore of -8.998 kcal/mol) and the original crystallographic pose was found to be 0.6224 Å. As shown in the **Fig.S1**, the docked position of the ligand closely aligns with the original ligand's position, evident from the comparable stacking configurations within the binding site. This strong agreement, both visually and in RMSD values, validates that our docking protocol can accurately predict the binding orientation of the actual substrate.

## Principal component analysis

Principal Component Analysis (PCA) was conducted to explore the essential motions of the 3CLpro-ligand complexes during MD simulations. PCA reduced the dimensionality of atomic positional data, identifying dominant collective motions that contribute to the protein's dynamics. The covariance matrix of the atomic fluctuations was constructed using the Cα atoms of the protein, with each element representing the covariance between atomic displacements along the trajectory. The matrix was diagonalized, and the resulting eigenvectors and eigenvalues represent the principal components (PCs) and their respective contribution to the overall motion. The first few PCs, capturing the largest variance, were analysed to determine the most relevant conformational changes. Trajectories projected onto these PCs provided insights into significant motions within the protein-ligand complexes. The GROMACS 2020.7 software was employed for PCA, and visualizations were generated using Origin.

## Free energy landscape analysis

Free Energy Landscape (FEL) analysis was performed to assess the conformational states of the SARS-CoV-2 3CLpro-ligand complexes over the course of the MD simulations. FEL provided a thermodynamic view of the system by mapping free energy as a function of selected reaction coordinates. In this study, RMSD and Rg were used as reaction coordinates to describe the conformational space. The FEL was constructed using the formula low-energy basins in the FEL correspond to stable conformational states, while high-energy regions indicate less favorable configurations. The GROMACS 2020.7 suite was used to generate FELs, and plots were visualized with Origin to provide a clear representation of the energy landscape.


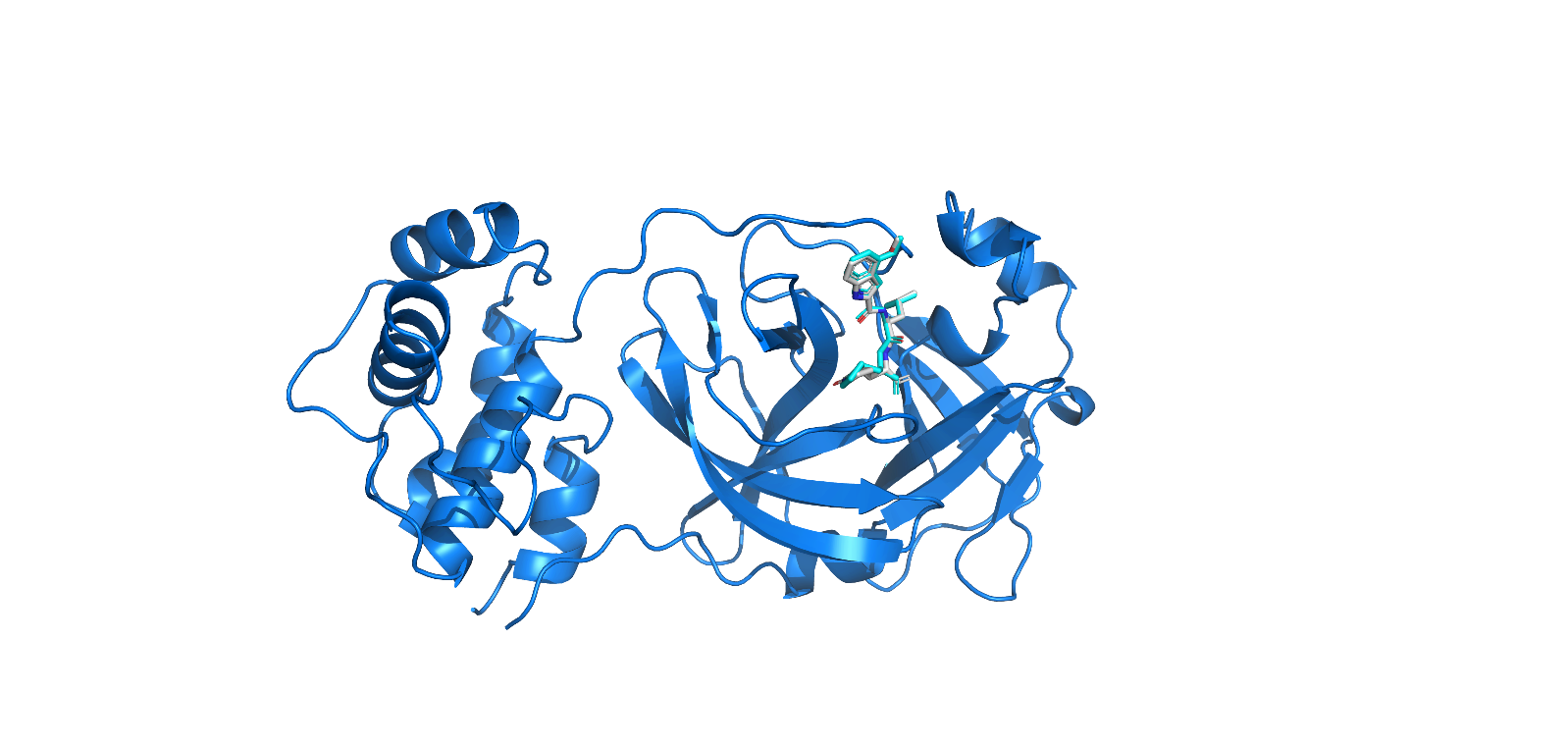

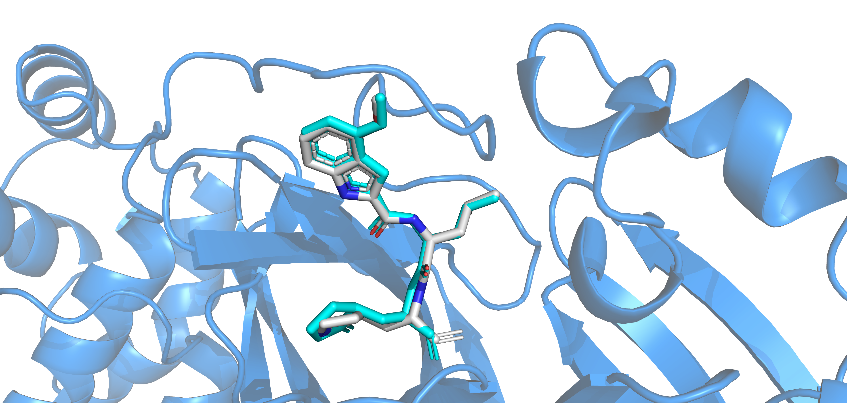


**Fig. S1** Alignment of redocked (blue) and crystallographic (white) ligand in 3CLpro active site validating docking precision.

**Table S1** The XP Gscore and MMGBSA energies of 44 top compounds and **S-217622**.

| Compound | Structure | XP GScore (kcal/mol) | MM/GBSA  *∆G_bind_* (kcal/mol) | Strain Energy  (kcal/mol) |
| --- | --- | --- | --- | --- |
| **1** |  | $-$8.45 | $-$71.32 | 7.838 |
| **2** |  | $-$8.21 | $-$61.37 | 10.936 |
| **3** |  | $-$8.55 | $-$52.47 | 4.617 |
| **4** |  | $-$8.59 | $-$70.62 | 4.478 |
| **5** |  | $-$7.57 | $-$71.18 | 5.355 |
| **6** |  | $-$8.82 | $-$50.32 | 4.675 |
| **7** |  | $-$9.24 | $-$63.31 | 3.470 |
| **8** |  | $-$9.10 | $-$63.46 | 2.051 |
| **9** |  | $-$9.34 | $-$61.90 | 2.473 |
| **10** |  | $-$10.05 | $-$60.93 | 4.513 |
| **11** |  | $-$9.17 | $-$76.38 | 3.672 |
| **12** |  | $-$9.34 | $-$70.91 | 4.236 |
| **13** |  | $-$8.29 | $-$66.54 | 4.719 |
| **14** |  | $-$9.25 | $-$63.32 | 7.172 |
| **15** |  | $-$10.13 | $-$65.10 | 4.841 |
| **16** |  | $-$8.47 | $-$71.42 | 6.536 |
| **17** |  | $-$7.82 | $-$60.64 | 6.107 |
| **18** |  | $-$9.74 | $-$62.64 | 3.564 |
| **19** |  | $-$8.76 | $-$70.24 | 6.233 |
| **20** |  | $-$7.21 | $-$70.27 | 3.106 |
| **21** |  | $-$6.99 | $-$70.49 | 4.215 |
| **22** |  | $-$7.57 | $-$62.77 | 3.915 |
| **23** |  | $-$7.78 | $-$70.96 | 9.191 |
| **24** |  | $-$7.26 | $-$68.56 | 2.476 |
| **25** |  | $-$5.34 | $-$63.97 | 21.117 |
| **26** |  | $-$8.24 | $-$67.16 | 3.751 |
| **27** |  | $-$4.72 | $-$67.47 | 5.861 |
| **28** |  | $-$6.82 | $-$64.99 | 2.029 |
| **29** |  | $-$7.05 | $-$61.66 | 5.015 |
| **30** |  | $-$8.80 | $-$60.77 | 13.993 |
| **31** |  | $-$8.16 | $-$61.19 | 4.619 |
| **32** |  | $-$6.38 | $-$66.64 | 0.769 |
| **33** |  | $-$9.21 | $-$64.39 | 3.644 |
| **34** |  | $-$7.25 | $-$58.30 | 11.528 |
| **35** |  | $-$7.77 | $-$64.71 | 7.360 |
| **36** |  | $-$8.01 | $-$62.49 | 3.857 |
| **37** |  | $-$8.04 | $-$62.15 | 1.091 |
| **38** |  | $-$9.23 | $-$59.29 | 6.604 |
| **39** |  | $-$8.18 | $-$56.96 | 4.019 |
| **40** |  | $-$8.69 | $-$62.34 | 1.467 |
| **41** |  | $-$5.88 | $-$63.69 | 3.712 |
| **42** |  | $-$7.93 | $-$62.13 | 4.201 |
| **43** |  | $-$6.52 | $-$61.76 | 1.837 |
| **44** |  | $-$8.38 | $-$60.78 | 4.563 |
| **S-217622** |  | $-$6.58 | $-$57.03 | 5.300 |

**Table S2** The ADMET properties of the 44 top compounds and **S-217622**.

| Compound | ABS | Water solubility | BBB | P-gp | LD50 (mg/kg) | Toxicity Class |
| --- | --- | --- | --- | --- | --- | --- |
| **1** | High | Moderately | Yes | Yes | 1000 | Ⅳ |
| **2** | High | Moderately | No | Yes | 540 | Ⅳ |
| **3** | High | Moderately | No | Yes | 800 | Ⅳ |
| **4** | High | Moderately | No | No | 1460 | Ⅳ |
| **5** | High | Moderately | No | Yes | 1240 | Ⅳ |
| **6** | High | Moderately | No | Yes | 200 | Ⅲ |
| **7** | High | Moderately | No | Yes | 1000 | Ⅳ |
| **8** | High | Moderately | No | Yes | 300 | Ⅲ |
| **9** | High | Moderately | No | Yes | 1460 | Ⅳ |
| **10** | Low | Moderately | Yes | No | 1600 | Ⅳ |
| **11** | High | Moderately | No | Yes | 1240 | Ⅳ |
| **12** | Low | Moderately | No | Yes | 1000 | Ⅳ |
| **13** | High | Moderately | No | Yes | 1000 | Ⅳ |
| **14** | High | Moderately | No | Yes | 572 | Ⅳ |
| **15** | High | Moderately | Yes | No | 1000 | Ⅳ |
| **16** | High | Moderately | No | Yes | 1646 | Ⅳ |
| **17** | High | Moderately | No | No | 4000 | V |
| **18** | High | Moderately | No | Yes | 1000 | Ⅳ |
| **19** | High | Soluble | No | Yes | 707 | Ⅳ |
| **20** | High | Moderately | No | Yes | 1460 | Ⅳ |
| **21** | High | Moderately | Yes | No | 1800 | Ⅳ |
| **22** | High | Moderately | Yes | No | 250 | Ⅲ |
| **23** | Low | Moderately | No | No | 1000 | Ⅳ |
| **24** | High | Moderately | No | Yes | 1460 | Ⅳ |
| **25** | High | Moderately | No | No | 560 | Ⅳ |
| **26** | High | Moderately | No | No | 1100 | Ⅳ |
| **27** | High | Moderately | No | Yes | 1500 | Ⅳ |
| **28** | High | Soluble | Yes | Yes | 1000 | Ⅳ |
| **29** | High | Soluble | No | Yes | 650 | Ⅳ |
| **30** | High | Soluble | No | Yes | 1000 | Ⅳ |
| **31** | High | Moderately | Yes | No | 2000 | Ⅳ |
| **32** | High | Moderately | No | Yes | 1500 | Ⅳ |
| **33** | High | Moderately | Yes | Yes | 3000 | V |
| **34** | High | Moderate | Yes | No | 1750 | Ⅳ |
| **35** | High | Moderate | Yes | No | 3000 | V |
| **36** | High | Moderate | No | No | 4000 | V |
| **37** | High | Moderate | Yes | No | 500 | Ⅳ |
| **38** | High | Soluble | No | Yes | 2300 | V |
| **39** | High | Moderately | Yes | No | 1500 | Ⅳ |
| **40** | High | Soluble | No | Yes | 1000 | Ⅳ |
| **41** | Low | Moderately | No | Yes | 1000 | Ⅳ |
| **42** | High | Moderately | Yes | No | 1500 | Ⅳ |
| **43** | High | Moderate | No | No | 1000 | Ⅳ |
| **44** | High | Moderate | No | Yes | 1000 | Ⅳ |
| **S-217622** | High | Moderate | No | Yes | 1000 | Ⅳ |

**Table S3** The toxicity properties of the44 top compounds and **S-217622**.

| Compound | Hepatotoxicity | Neurotoxicity | Respiratory toxicity | Carcinogenicity | Immunotoxicity | Mutagenicity |
| --- | --- | --- | --- | --- | --- | --- |
| **1** | Inactive (0.90) | active (0.64) | active (0.88) | Inactive (0.53) | active  (0.71) | Inactive (0.70) |
| **2** | Inactive (0.76) | active (0.60) | active (0.66) | Inactive (0.70) | Inactive (0.61) | Inactive (0.61) |
| **3** | Inactive (0.84) | active (0.51) | active (0.77) | Inactive (0.56) | active  (0.87) | Inactive (0.64) |
| **4** | Inactive (0.51) | active (0.91) | active (0.79) | active  (0.57) | Inactive (0.89) | Inactive (0.59) |
| **5** | Inactive (0.66) | active (0.75) | active (0.85) | Inactive (0.58) | Inactive (0.90) | Inactive (0.58) |
| **6** | Inactive (0.72) | active (0.66) | active (0.72) | Inactive (0.59) | Inactive (0.96) | Inactive (0.56) |
| **7** | active (0.53) | active (0.61) | active (0.60) | Inactive (0.62) | active  (0.85) | Inactive (0.60) |
| **8** | Inactive (0.54) | active (0.82) | active (0.80) | Inactive (0.83) | Inactive (0.94) | Inactive (0.62) |
| **9** | Inactive (0.64) | active (0.88) | active (0.75) | active  (0.68) | Inactive (0.99) | Inactive (0.58) |
| **10** | active (0.60) | active (0.76) | active (0.70) | active  (0.54) | active  (0.97) | active (0.58) |
| **11** | Inactive (0.65) | active (0.80) | active (0.89) | Inactive (0.61) | Inactive (0.91) | Inactive (0.62) |
| **12** | Inactive (0.50) | active (0.50) | active (0.60) | Inactive (0.62) | Inactive (0.87) | Inactive (0.61) |
| **13** | Inactive (0.87) | Inactive (0.57) | active (0.66) | Inactive (0.64) | Inactive (0.95) | Inactive (0.86) |
| **14** | Inactive (0.79) | active (0.66) | active (0.76) | Inactive (0.63) | Inactive (0.55) | Inactive (0.68) |
| **15** | active (0.51) | active (0.65) | active (0.76) | active  (0.58) | Inactive (0.58) | active (0.74) |
| **16** | Inactive (0.83) | active (0.83) | active (0.82) | Inactive (0.72) | Inactive (0.86) | Inactive (0.67) |
| **17** | Inactive (0.83) | Inactive (0.58) | active (0.65) | Inactive (0.62) | Inactive (0.99) | Inactive (0.74) |
| **18** | Inactive (0.74) | active (0.60) | active (0.66) | Inactive (0.59) | active  (0.94) | Inactive (0.61) |
| **19** | Inactive (0.87) | active (0.50) | active (0.67) | Inactive (0.65) | Inactive (0.95) | Inactive (0.59) |
| **20** | Inactive (0.52) | active (0.84) | active (0.81) | active  (0.62) | Inactive (0.97) | Inactive (0.61) |
| **21** | Inactive (0.82) | Inactive (0.66) | active (0.57) | Inactive (0.56) | Inactive (0.87) | Inactive (0.76) |
| **22** | Inactive (0.71) | active (0.57) | active (0.78) | Inactive (0.67) | active  (0.99) | Inactive (0.69) |
| **23** | active (0.52) | active (0.65) | active (0.63) | active  (0.59) | Inactive (0.99) | Inactive (0.73) |
| **24** | Inactive (0.56) | active (0.90) | active (0.75) | active  (0.73) | Inactive (0.99) | Inactive (0.62) |
| **25** | Inactive (0.60) | active (0.88) | active (0.86) | Inactive (0.56) | Inactive (0.97) | Inactive (0.51) |
| **26** | Inactive (0.81) | Inactive (0.54) | active (0.55) | Inactive (0.57) | Inactive (0.96) | Inactive (0.75) |
| **27** | Inactive (0.78) | active (0.68) | active (0.66) | Inactive (0.62) | Inactive (0.99) | Inactive (0.69) |
| **28** | Inactive (0.87) | active (0.52) | active (0.83) | Inactive (0.60) | active  (0.56) | Inactive (0.70) |
| **29** | Inactive (0.60) | active (0.80) | active (0.87) | Inactive (0.52) | active  (0.98) | Inactive (0.52) |
| **30** | active (0.57) | active (0.55) | active (0.51) | active  (0.58) | Inactive (0.99) | Inactive (0.56) |
| **31** | Inactive (0.76) | active (0.51) | active (0.52) | Inactive (0.62) | Inactive (0.88) | Inactive (0.73) |
| **32** | Inactive (0.79) | active (0.67) | active (0.66) | Inactive (0.63) | Inactive (0.99) | Inactive (0.70) |
| **33** | Inactive (0.66) | Inactive (0.55) | Inactive (0.50) | Inactive (0.59) | Inactive (0.99) | Inactive (0.68) |
| **34** | Inactive (0.79) | active (0.50) | active (0.54) | Inactive (0.62) | Inactive (0.91) | Inactive (0.74) |
| **35** | Inactive (0.68) | active (0.55) | active (0.70) | Inactive (0.52) | Inactive (0.87) | Inactive (0.66) |
| **36** | Inactive (0.51) | Inactive (0.81) | Inactive (0.85) | Inactive (0.62) | Inactive (0.62) | Active (0.86) |
| **037** | active (0.53) | active (0.71) | active (0.76) | active  (0.55) | Inactive (0.91) | active (0.74) |
| **38** | Inactive (0.89) | active (0.52) | active (0.68) | Inactive (0.65) | Inactive (0.92) | Inactive (0.71) |
| **39** | Inactive (0.78) | active (0.62) | Inactive (0.53) | Inactive (0.67) | Inactive (0.99) | Inactive (0.79) |
| **40** | Inactive (0.78) | active (0.54) | active (0.72) | Inactive (0.59) | active  (0.92) | Inactive (0.66) |
| **41** | Inactive (0.55) | Inactive (0.66) | active (0.53) | Inactive (0.64) | active  (0.99) | Inactive (0.61) |
| **42** | Inactive (0.77) | active (0.53) | Inactive (0.50) | Inactive (0.66) | Inactive (0.98) | Inactive (0.79) |
| **43** | Inactive (0.56) | active (0.56) | active (0.56) | active  (0.54) | Inactive (0.52) | Inactive (0.63) |
| **44** | Inactive (0.57) | active (0.70) | active (0.80) | Inactive (0.59) | Inactive (0.99) | Inactive (0.59) |
| **S-217622** | Inactive (0.55) | active (0.89) | active (0.83) | Inactive (0.58) | active  (0.92) | active (0.57) |

**Table S4** Inhibition of SARS-CoV-2 3CLpro Activity by 44 top compounds

| compound | Inhibition %  SARS-CoV-2 3CLpro  @ 50 $\mu$M | compound | Inhibition %  SARS-CoV-2 3CLpro  @ 50 $\mu$M |
| --- | --- | --- | --- |
| **1** | 85.4 | **23** | 33.0 |
| **2** | 13.7 | **24** | 41.0 |
| **3** | 60.3 | **25** | $-$13.4 |
| **4** | 62.1 | **26** | 42.3 |
| **5** | 84.0 | **27** | 29.8 |
| **6** | 0.15 | **28** | $-$8.6 |
| **7** | 20.0 | **29** | 15.2 |
| **8** | $-$9.0 | **30** | $-$21.2 |
| **9** | 3.8 | **31** | 50.0 |
| **10** | 78.0 | **32** | 15.0 |
| **11** | 49.0 | **33** | 42.0 |
| **12** | 24.3 | **34** | 78.3 |
| **13** | 11.9 | **35** | 13.3 |
| **14** | $-$82.0 | **36** | 81.9 |
| **15** | 61.0 | **37** | 24.7 |
| **16** | $-$2.5 | **38** | $-$10.8 |
| **17** | 58.0 | **39** | 53.9 |
| **18** | $-$105.0 | **40** | 5.4 |
| **19** | 12.3 | **41** | $-$25.1 |
| **20** | $-$3.1 | **42** | $-$12.3 |
| **21** | 32.8 | **43** | 84.8 |
| **22** | 61.0 | **44** | 38.2 |
| **S-217622** | 99.2 |  |  |

a: % inhibition = Kinase activity inhibited, determined at 50 μM of compound concentration.
